# Peritonitis Secondary to Uncommon Gram-Negative Coccobacillus Transmitted From a Cat in a Patient on Peritoneal Dialysis

**DOI:** 10.1177/2324709619895165

**Published:** 2019-12-14

**Authors:** Sreedhar Adapa, Srikanth Naramala, Bhaskar Reddy Madhira, Vijay Gayam, Prem Sahasranam, Venu Madhav Konala

**Affiliations:** 1 Kaweah Delta Medical Center, Visalia, CA, USA; 2 Adventist Medical Center, Hanford, CA, USA; 3 SUNY Upstate Medical University, Syracuse, NY, USA; 4 Interfaith Medical Center, Brooklyn, NY, USA; 5 Hanford Family Medicine Residency Program, Hanford, CA, USA; 6 Ashland Bellefonte Cancer Center, Ashland, KY, USA

**Keywords:** *Pasteurella multocida*, cats, peritoneal dialysis, peritonitis, gram-negative coccobacilli

## Abstract

Peritonitis caused by gram-negative organisms is a significant complication encountered in patients undergoing peritoneal dialysis and is associated with high morbidity and mortality. There has been recognition of peritonitis caused by uncommon organisms because of improved microbiological detection techniques. In this article, we report a rare case of peritonitis caused by *Pasteurella multocida*. We present a 58-year-old male on peritoneal dialysis with fever and abdominal pain. The peritoneal fluid was cloudy, and the analysis was consistent with peritonitis. The peritoneal fluid culture grew *Pasteurella multocida*. The patient was treated with a 3-week course of intraperitoneal ceftazidime, which resulted in the resolution of infection with the salvation of the peritoneal dialysis catheter. Patient education plays a very critical role in the prevention of peritonitis from *Pasteurella multocida*, particularly if patients have pets at home. The domestic pets should be kept away from the dialysis equipment and should not be allowed into the room during dialysis treatment. Incorporating the education in handing pets during the training session is the key aspect.

## Introduction

Peritonitis caused by gram-negative organisms is a significant complication encountered in patients undergoing peritoneal dialysis (PD) and is often associated with high morbidity and mortality.^1^ *Pasteurella multocida* is an aerobic and facultative anaerobic gram-negative coccobacillus, which is a normal commensal of oropharynx of many animals but is predominant in domestic pets like cats and dogs.^2^ It was first identified in 1878 by Perroncito and was named after Louis Pasteur in 1880 who described this bacterium in diseased birds.^3^ This bacterium is found in the oropharynx of 30% of live stockbreeders.^4^ Infection with bacteria is rare in humans but commonly occurs in immunocompromised patients. It is susceptible to most common antibiotics.

## Case Report

A 58-year-old male on PD presented to the outpatient dialysis clinic with a chief complaint of fever, abdominal pain, and cloudy effluent for 1-day duration. He denied any touch contamination. The patient has been on PD for 4 months. He has been working as a supervisor and it was a desk job. The patient denied any history of smoking, alcohol intake, or illicit drug use. The patient is married and has cats as domestic pets. The patient admitted that the cats were present in the room while he was performing PD. As per the patient, he always washed his hands while accessing PD equipment. The patient had not witnessed that the cats licked, bitten, or scratched the PD equipment. There was no leakage witnessed in the PD equipment. Past medical history is signification for polycystic kidney disease, diabetes, hypertension, hyperlipidemia, hyperparathyroidism, hypothyroidism, seizure disorder, end-stage renal disease secondary to polycystic kidney disease on PD. His home medications include atorvastatin 40 mg daily at night time, ferric citrate 210 mg one tablet 3 times a day with meals, sevelamer 800 mg 3 tablets 3 times a day with meals, omega 3 fatty acid 1 capsule daily, lactulose 20 g daily as needed for constipation, levetiracetam 500 mg twice a day, levothyroxine 100 µg daily, metoprolol 25 mg twice a day, cinacalcet 30 mg daily, and renal vitamin 1 tablet daily.

The vital signs on presentation were the temperature of 100.2°F, pulse rate of 74 beats per minute, respiratory rate of 18 breaths per minute, and blood pressure of 143/82 mm Hg. Physical examination reveals diffuse abdominal tenderness with a PD catheter in the right lower quadrant. There is neither exit site drainage nor redness along the tunnel of the catheter. The rest of the physical examination was unremarkable.

Laboratory data revealed white blood cell (WBC) count 5500/mm^3^, hemoglobin 11.3 g/dL, platelet count 260 000/mm^3^, sodium 142 mmol/L, potassium 4.2 mmol/L, bicarbonate 21 mmol/L, blood urea nitrogen 51 mg/dL, creatinine 13.8 mg/dL, and albumin 3.5 g/dL. The peritoneal fluid effluent revealed peritoneal fluid WBC 4576 cells/µL with 94% neutrophils. Peritoneal fluid gram stain revealed >100 WBC count and no organisms seen. The patient was started on empiric treatment with intraperitoneal (IP) vancomycin and ceftazidime for peritonitis. Peritoneal fluid culture grew *P multocida* in both aerobic and anaerobic bottles. The sensitivities of *P multocida* are listed in Table 1.

**Table 1.** Sensitivities of *Pasteurella multocida*.

Antibiotic	Minimum Inhibitory Concentration	Sensitivity Result (S = Sensitive, R = Resistant)
Ampicillin	0.5 µg/mL	S
Ceftazidime	≤0.03 µg/mL	S
Chloramphenicol	1 µg/mL	S
Levofloxacin	<0.03 µg/mL	S
Penicillin	0.25 µg/mL	S
Trimethoprim/sulfamethoxazole	0.25/4.75 µg/mL	S

Antibiotic therapy subsequently tailored to IP ceftazidime as per sensitivities for 3 weeks. The repeat peritoneal fluid cultures after finishing antibiotic course yielded no bacterial growth, and the PD catheter was salvaged. The patient was counseled to prevent pets from contaminating the dialysis supplies or pets from entering the treatment room.

## Discussion

Peritonitis is an important and serious complication often resulting in PD catheter loss and change in dialysis modality. When the infection does not respond to the routine treatment, infection from unusual organisms should be suspected including zoonosis. There has been increased recognition of peritonitis caused by rare organisms like *P multocida*, which is related to close contact with pets.

Pets are an integral part of many households and share close bonding with the owners. Pets play a crucial role in coping with stress and provide psychological support. The close contact with the pets poses potential health risks like infections, allergies, and injuries sustained from bites, scratches, and attacks. Close contact with pets should prompt the health care provider to suspect zoonosis.^5^

The first case of *P multocida* PD-associated peritonitis was reported in 1987.^6^ Peritonitis from this organism has been reported infrequently but has been recognized as a significant cause. The increase in the number of cases reported might be attributed to the increased pet breeding at home. We summarized all the cases listed as *P multocida* peritonitis on literature review from PubMed in Table 2. Cats cause most of the PD-related peritonitis from *P multocida*, accounting for more than 90%of the cases.^2^ Mode of transmission of this zoonotic organism is through the licks, bites, and scratches of the pets.^2^ Transmission can also be from contamination of the dialysis machine and tubes. Another possible way of transmission is hand contamination from the patient’s oropharyngeal colonization of *P multocida*.^4^

**Table 2.** Summary of All the Cases Listed as *Pasteurella multocida* Peritonitis With Patients on Dialysis as per PubMed Review of Literature.

Author	Year	Age/Gender	Duration (Months)	Dialysis Mode	Leakage	Dialysate Culture	Animal Exposure	Treatment	Outcome
Paul and Rostand^6^	1987	55/female	4	CCPD	Present	Positive	Cat bite/scratch	Gentamicin	Improved
Frankel and Cassidy^7^	1991	55/male	15	CAPD	Absent	Positive	Cat exposure	Gentamicin, ciprofloxacin	Improved
London and Bottone^8^	1991	54/male	6	CCPD	Present	Positive	Cat bite	Cefazolin, gentamicin	Improved
Elsey et al^9^	1991	25/male	≤24	CCPD	Absent	Positive	Cat exposure	Cephradine, gentamicin	Improved
Kitching et al^10^	1996	75/male	6	CAPD	Present	Positive	Cat bite	Cefamandole	Improved
Uribarri et al^11^	1996	42/female	108	CCPD	Present	Positive	Cat bite	Penicillin, gentamicin	Improved
Loghman-Adham^12^	1997	12/female	7	CCPD	Present	Positive	Cat bite	Cephapirin, gentamicin	Improved
Mackay et al^13^	1997	73/male	12	CAPD	Absent	Positive	Cat exposure	Ceftazidime	Improved
Joh et al^14^	1998	55/male	12	CCPD	Present	Positive	Cat bite	Ampicillin/sulbactam, gentamicin	Improved
Musio and Tiu^15^	1998	46/female	7	CCPD	Absent	Positive	Cat exposure	Piperacillin, ciprofloxacin	Improved
Hamai et al^16^	1999	49/male	4	CCPD	Present	Positive	Cat exposure	Cefazolin, tobramicin	Improved
Chadha and Warady^17^	1999	16/male	60	CCPD	Present	Positive	Cat bite	Ticarcillin, tobramicin	
Van Langenhove et al^18^	2000	22/female	12	CCPD	Present	Positive	Cat scratch	Amikacin, ciprofloxacin	Improved
Martinez et al^19^	2000	46/female	NA	CCPD	NA	Positive	NA	Ceftazidime	NA
Kanaan et al^20^	2002	24/female	7	CCPD	Absent	Positive	Cat exposure	Ciprofloxacin	Improved
Sillery et al^21^	2004	48/female	36	CAPD	Absent	Positive	Cat exposure	Cefazolin, gentamicin, ampicillin	Improved
Cooke et al^22^	2004	73/female	8 and 12	CAPD	Present	Positive	Cat bite	Gentamicin, ciprofloxacin	Improved
Mat et al^23^	2005	52/male	3	CCPD	Present	Positive	Cat exposure	Cefazolin, amikacin	Improved
Malik et al^24^	2005	21/female	36	CCPD	Present	Positive	Cat bite	Gentamicin, cefazolin piperacillin/tazobactam	Improved
Malik et al^24^	2005	58/male	12	CCPD	Present	Positive	Cat bite	Gentamicin	Improved
Olea et al^25^	2006	46/female	24	CCPD	Absent	Positive	Cat exposure	Ceftazidime	Improved, catheter removed
Antony and Oglesby^26^	2007	48/female	NA	CAPD	Absent	Positive	Dog exposure	Cefazolin, gentamicin	Improved
Rondon-Berrios and Trevejo-Nunez^4^	2010	38/male	60	CCPD	Present	Positive	Cat exposure	Piperacillin/tazobactam, ampicillin, levofloxacin	Improved, catheter removed
Mugambi and Ullian^27^	2010	36/female	NA	CCPD	Absent	Positive	Cat exposure	Gentamicin, ciprofloxacin	Improved, catheter removed
Satomura et al^28^	2010	58/male		CCPD	Absent	Positive	Cat exposure	Cefazolin, levofloxacin	Improved
Nishina et al^29^	2011	45/male	84	CCPD	Present	Positive	Cat exposure	Ceftazidime, levofloxacin	Improved
Weiss and Panesar^30^	2012	57/male	1	CAPD	Absent	positive	Cat and dog exposure	Vancomycin, ceftazidime	Improved
Sol et al^31^	2013	7/female	24	NIPD	Present	Positive	Cat exposure	Ampicillin	Improved
Kim et al^2^	2014	25/female	24	CAPD	Absent	Positive	Cat exposure	Cefazolin, gentamicin	Improved
Dresselaars et al^32^	2014	62/female	37	CAPD	Absent	Positive	Cat exposure	Cefalothin, ciprofloxacin	Improved
Poliquin et al^33^	2015	28/female	1	CAPD	Present	Positive	Cat bite	Ceftazidime	Improved
Poliquin et al^33^	2015	37/male	15	CAPD	Absent	Positive	Cat bite	Cefazolin	Improved
Poliquin et al^33^	2015	41/male	18	CAPD	NA	Positive	Cat bite	Cefazolin	Improved
Poliquin et al^33^	2015	51/female	7	CAPD	Absent	Positive	Cat exposure	Amoxicillin-clavulanic acid	Improved
Poliquin et al^33^	2015	37/female	132	CAPD	Absent	Positive	Cat exposure	Ceftriaxone, amoxicillin	Improved, catheter removed
Poliquin et al^33^	2015	59/female	36	CAPD	Absent	Positive	Cat exposure	Ceftazidime	Improved
Poliquin et al^33^	2015	69/female	1	CAPD	NA	Positive	Cat bite	Ceftazidime	Improved
Giron et al^34^	2017	72/male	24	CCPD	Present	Positive	Cat bit	Ceftazidime	Improved

Abbreviations: CCPD, continuous cyclic peritoneal dialysis; CAPD, continuous ambulatory peritoneal dialysis; NA, not available; NIPD, nocturnal intermittent peritoneal dialysis.

In an analysis of 124 patients on PD done by Broughton et al,^5^ 12 different zoonotic organisms caused peritonitis and animals were involved in 24% of the cases. Catheter loss occurred in 27% of the patients, and the overall mortality was 24%. *Pasteurella* species peritonitis was reported in 24 patients, and cats were involved in 21 cases. Other pets involved were dogs and hamsters.^5^

This bacterium is normal commensal in 50% to 90% of cats and 50% to 66% of the dogs in their oral cavity. It is also present in the claws of 20% of the cats.^35^ As per Nishina et al, two thirds of the patients presented with peritonitis within 12 months of initiation of PD.^29^ Most of the reported cases of peritonitis associated with this pathogen were due to close contact with cats or puncturing of the dialysis tubing by cats.

The bacterium is implicated in soft tissue infections, septic arthritis, pneumonia, and endocarditis in humans.^36^ Invasive infections are more common in immunocompromised patients including cirrhosis, alcoholism, malignancy, diabetes mellitus, human immune virus deficiency infection, chronic pulmonary disease, and chronic kidney disease.^37^ Life-threatening infections reported including infective endocarditis and sepsis.^36^

Patients doing continuous ambulatory PD are less prone to develop peritonitis from *P multocida* compared with patients on continuous cyclic PD because of fewer chances for the pets to come in contact with the dialysis bags and tubing. The patients who had peritonitis from *P multocida* were either on continuous cyclic PD or nocturnal intermittent PD in most of the cases reported.^2,29^ The pets, mostly cats, play with the longer dialysis tubes.^2^ The dialysis tube trembling and the pump sound on the cycler may be an attractive toy for the cats.^29^

Patients who are initiated on PD should be questioned about the pets they own or the intention of owning the pets. Incorporating the education on proper handling of the pets during training sessions plays a very critical role in the prevention of peritonitis from *P multocida*. Regular care of the pets, careful hand washing, proper disposal of animal waste, and placing barriers that limit the access of the pet to the dialysis equipment are the interventions that showed a significant impact on the behavior of patients toward pets. Abebe et al demonstrated this at a single dialysis center resulting in the resolution of pets-related peritonitis.^38^ Most reported cases of peritonitis from *P multocida* mostly by contact from pets reiterates the importance of personal hygiene.

*Pasteurella multocida* peritonitis symptoms are evident as early as 24 hours with fever, cloudy effluent, and severe abdominal pain.^4^ Dialysis cultures are usually positive, with negative gram stain and blood cultures.^4^ *P multocida* organisms produce a characteristic mousy odor and grow on blood or chocolate agar at 37°C.^39^ Molecular techniques like DNA sequencing and 16S rRNA gene polymerase chain reaction are used for rapid identification and characterization of *P multocida* with 16S rRNA gene polymerase chain reaction most commonly used at present.^40^

Symptoms improve rapidly within 48 to 96 hours after initiation of treatment.^4^ *P multocida* is susceptible to most of the antibiotics, which covers gram-negative bacteria.^29^ The IP antibiotic treatment for 3 weeks is sufficient.^2^ The most frequently used antibiotic is gentamicin.^29^ *Pasteurella* can produce β-lactamases and can be penicillin resistant, and hence, ampicillin/sulbactam, amoxicillin/clavulanate, and piperacillin/tazobactam are recommended for the treatment.^29^ There is a high likelihood of PD catheter salvage, with catheter removal in only 11% of cases. There has been no mortality reported with *P multocida* peritonitis.^34^

## Conclusion

Education plays a very critical role in the prevention of peritonitis from *P multocida*, particularly if patients have pets at home. Hand washing, regular pet care, proper handling of animal waste, and adding barriers that will prevent pets from accessing the PD equipment are the potential interventions. Preventing the pets from entering the room while making connections and treatment also alleviates the risk of peritonitis from *P multocida*. Incorporating education in handing pets during the training session is a crucial aspect.

## References

[1] Adapa S Naramala S Boken D Moreno A Konala VM. Peritonitis from anaerobic gram-positive cocci likely due to translocation of bacteria from gut in a patient undergoing peritoneal dialysis. Cureus. 2019;11:e6060. 31827990 10.7759/cureus.6060 PMC6890156

[2] Kim I Kim YW Chung S Yoon HE Shin SJ. Cat-induced *Pasteurella multocida* peritonitis in continuous ambulatory peritoneal dialysis. Kidney Res Clin Pract. 2014;33:65-67. 26877951 10.1016/j.krcp.2013.11.003 PMC4714178

[3] Kubatzky KF. *Pasteurella multocida* and immune cells. Curr Top Microbiol Immunol. 2012;361:53-72. 22427181 10.1007/82_2012_204

[4] Rondon-Berrios H Trevejo-Nunez GJ. Pets or pest: peritoneal dialysis-related peritonitis due to *Pasteurella multocida*. J Microbiol Immunol Infect. 2010;43:155-158. 20457433 10.1016/S1684-1182(10)60024-2

[5] Broughton A Verger C Goffin E. Pets-related peritonitis in peritoneal dialysis: companion animals or Trojan horses? Semin Dial. 2010;23:306-316. 20636924 10.1111/j.1525-139X.2010.00726.x

[6] Paul RV Rostand SG. Cat-bite peritonitis: *Pasteurella multocida* peritonitis following feline contamination of peritoneal dialysis tubing. Am J Kidney Dis. 1987;10:318-319. 3661553 10.1016/s0272-6386(87)80029-x

[7] Frankel AH Cassidy MJ. *Pasteurella multocida* peritonitis in CAPD: beware of the cats. Perit Dial Int. 1991;11:184-185. 1854879

[8] London RD Bottone EJ. *Pasteurella multocida*: zoonotic cause of peritonitis in a patient undergoing peritoneal dialysis. Am J Med. 1991;91:202-204. 1867248 10.1016/0002-9343(91)90018-s

[9] Elsey RM Carson RW DuBose TD Jr. *Pasteurella multocida* peritonitis in an HIV-positive patient on continuous cycling peritoneal dialysis. Am J Nephrol. 1991;11:61-63. 2048580 10.1159/000168274

[10] Kitching AR Macdonald A Hatfield PJ. *Pasteurella multocida* infection in continuous ambulatory peritoneal dialysis. N Z Med J. 1996;109:59. 8598943

[11] Uribarri J Bottone EJ London RD. *Pasteurella multocida* peritonitis: are peritoneal dialysis patients on cyclers at increased risk? Perit Dial Int. 1996;16:648-649. 8981542

[12] Loghman-Adham M. *Pasteurella multocida* peritonitis in patients undergoing peritoneal dialysis. Pediatr Nephrol. 1997;11:353-354. 9203191 10.1007/s004670050295

[13] MacKay K Brown L Hudson F. *Pasteurella multocida* peritonitis in peritoneal dialysis patients: beware of the cat. Perit Dial Int. 1997;17:608-610. 9655162

[14] Joh J Padmanabhan R Bastani B *Pasteurella multocida* peritonitis following cat bite of peritoneal dialysis tubing. With a brief review of the literature. Am J Nephrol. 1998;18:258-259. 9627047 10.1159/000013330

[15] Musio F Tiu A. *Pasteurella multocida* peritonitis in peritoneal dialysis. Clin Nephrol. 1998;49:258-261. 9582558

[16] Hamai K Imai H Ohtani H, et al Repeated cat-associated peritonitis in a patient on automated nocturnal intermittent peritoneal dialysis. Clin Exp Nephrol. 1999;3:59-61.

[17] Chadha V Warady BA. *Capnocytophaga canimorsus* peritonitis in a pediatric peritoneal dialysis patient. Pediatr Nephrol. 1999;13:646-648. 10502119 10.1007/s004670050673

[18] Van Langenhove G Daelemans R Zachee P Lins RL *Pasteurella multocida* as a rare cause of peritonitis in peritoneal dialysis. Nephron. 2000;8:283-284. 10.1159/000045676 10867548

[19] Martinez JRB Lleti MS Fuster AV Belles CP Serrano MG Abdominal infection by *Pasteurella* spp. A report of 3 cases [in Spanish]. Rev Clin Esp. 2000;200:139-142. 10804759

[20] Kanaan N Gavage P Janssens M Avesani V Gigi J Goffin E. *Pasteurella multocida* in peritoneal dialysis: a rare cause of peritonitis associated with exposure to domestic cats. Acta Clin Belg. 2002;57:254-256. 12534132 10.1179/acb.2002.050

[21] Sillery J Hargreaves J Marin P Lerma E Kuznia C Abbe C. *Pasteurella multocida* peritonitis: another risk of animal-assisted therapy. Infect Control Hosp Epidemiol. 2004;25:5-6. 14756211 10.1086/503486

[22] Cooke FJ Kodjo A Clutterbuck EJ Bamford KB. A case of *Pasteurella multocida* peritoneal dialysis-associated peritonitis and review of the literature. Int J Infect Dis. 2004;8:171-174. 15109592 10.1016/j.ijid.2003.10.004

[23] Mat O Moenens F Beauwens R, et al Indolent *Pasteurella multocida* peritonitis in a CCPD patient. 25 years of “cat-bite peritonitis”: a review. Perit Dial Int. 2005;25:88-90. 15770931

[24] Malik A Al Aly Z Mailey KS Bastani B. *Pasteurella multocida* peritoneal dialysis-associated peritonitis: a report of two cases and review of the literature. J Nephrol. 2005;18:791-793. 16358243

[25] Olea T Hevia C Bajo MA del Peso G Selgas R. *Pasteurella multocida* and *Candida albicans* peritonitis [in Spanish]. Nefrologia. 2006;26:136-138. 16649436

[26] Antony SJ Oglesby KA. Peritonitis associated with *Pasteurella multocida* in peritoneal dialysis patients—case report and review of the literature. Clin Nephrol. 2007;68:52-56. 17703837 10.5414/cnp68052

[27] Mugambi SM Ullian ME. Bacteremia, sepsis, and peritonitis with *Pasteurella multocida* in a peritoneal dialysis patient. Perit Dial Int. 2010;30:381-383. 20424206 10.3747/pdi.2009.00186

[28] Satomura A Yanai M Fujita T, et al Peritonitis associated with *Pasteurella multocida*: molecular evidence of zoonotic etiology. Ther Apher Dial. 2010;14:373-376. 20609195 10.1111/j.1744-9987.2009.00788.x

[29] Nishina M Yanagi H Koizumi M, et al *Pasteurella multocida* peritonitis associated with a cat in a peritoneal dialysis patient using an automated cycler device. CEN Case Rep. 2012;1:73-76. 28509064 10.1007/s13730-012-0016-3 PMC5411526

[30] Weiss GA Panesar M. *Pasteurella multocida* peritonitis with bacteremia on initiation of peritoneal dialysis. Perit Dial Int. 2012;32:363-364. 22641748 10.3747/pdi.2011.00191 PMC3525433

[31] Sol PM van de Kar NC Schreuder MF. Cat induced *Pasteurella multocida* peritonitis in peritoneal dialysis: a case report and review of the literature. Int J Hyg Environ Health. 2013;216:211-213. 22572039 10.1016/j.ijheh.2012.04.003

[32] Dresselaars HF Zwart B Pettersson AM Rijnsburger MC Ho-dac-Pannekeet MM. Peritoneal dialysis-associated peritonitis of zoonotic origin, when minor gets major. Neth J Med. 2014;72:551-553. 26219763

[33] Poliquin PG Lagacé-Wiens P Verrelli M Allen DW Embil JM. Pasteurella species peritoneal dialysis-associated peritonitis: household pets as a risk factor. Can J Infect Dis Med Microbiol. 2015;26:52-55. 25798157 10.1155/2015/389467 PMC4353272

[34] Giron FF Martín JS Gómez ER, et al Simultaneous *Streptococcus canis* and *Pasteurella multocida* peritonitis in a peritoneal dialysis patient. Perit Dial Int. 2017;37:483-484. 28676516 10.3747/pdi.2016.00286

[35] Weber DJ Wolfson JS Swartz MN Hooper DC. *Pasteurella multocida* infections. Report of 34 cases and review of the literature. Medicine (Baltimore) 1984;63:133-154. 6371440

[36] Wallace JA Hussain J Unzueta A Morelli G. *Pasteurella multocida* bacteremia and peritonitis in a patient with cirrhosis: a life-threatening case from a prick of a cactus. J Investig Med High Impact Case Rep. 2017;5:2324709617726103. 10.1177/2324709617726103 PMC5580853 28890902

[37] Gunathilake R Verma A Caffery M Sowden S. *Pasteurella multocida* peritonitis after cat scratch in a patient with cirrhotic ascites. Infect Dis Rep. 2015;7:5937. 26294953 10.4081/idr.2015.5937 PMC4508538

[38] Abebe M Laveglia C George S Wadhwa NK. Pet-related peritonitis and its prevention in peritoneal dialysis: a case study. Perit Dial Int. 2014;34:466-468. 24991056 10.3747/pdi.2013.00054 PMC4079496

[39] Pena D Santana Y Lara JP Gonzalez E Khaja M. Multiorgan failure and refractory lactic acidosis due to *Pasteurella multocida* septicemia in a patient with no animal exposure. Case Rep Infect Dis. 2018;2018:2574184. 29765783 10.1155/2018/2574184 PMC5885493

[40] Wilson BA Ho M. *Pasteurella multocida*: from zoonosis to cellular microbiology. Clin Microbiol Rev. 2013;26:631-655. 23824375 10.1128/CMR.00024-13 PMC3719492

